# The Emerging Role of Peroxyacetic Acid in Water and Wastewater Treatment: Degradation of Pharmaceuticals, Microplastics, and Other Micropollutants

**DOI:** 10.3390/molecules31101748

**Published:** 2026-05-20

**Authors:** Patrycja Zawiślak, Justyna Kapelewska, Izabela Ryza, Joanna Karpińska, Urszula Kotowska

**Affiliations:** 1 Doctoral School, University of Bialystok, Ciolkowskiego 1K St., 15-245 Bialystok, Poland; p.zawislak@uwb.edu.pl (P.Z.); i.ryza@uwb.edu.pl (I.R.); 2 Department of Analytical and Inorganic Chemistry, Faculty of Chemistry, University of Bialystok, Ciolkowskiego 1K St., 15-245 Bialystok, Poland; j.kapelewska@uwb.edu.pl (J.K.); joasia@uwb.edu.pl (J.K.)

**Keywords:** PAA, advanced oxidation processes, contaminants of emerging concern, chemical activators, physical activators

## Abstract

Conventional wastewater treatment systems cannot effectively eliminate micropollutants such as contaminants of emerging concern (CECs). These compounds, even at trace levels, are persistent or pseudo-persistent, bioaccumulative, and potentially harmful to ecosystems and human health. Advanced oxidation processes (AOPs), based on the in situ generation of highly reactive oxygen species, have emerged as promising solutions. Peroxyacetic acid (PAA) has gained attention due to its strong oxidizing capacity, broad antimicrobial activity, environmentally benign by-products, and compatibility with different activation methods. This review provides an updated and integrated synthesis of recent advances in PAA-based AOPs for the degradation of major CEC groups, including pharmaceuticals, personal care products, pesticides, and industrial chemicals, as well as for the oxidative modification of microplastics (MPs). The review discusses several strategies for PAA activation and critically discusses removal efficiency, underlying mechanisms, and current limitations, emphasizing the gap between pollutant transformation and complete mineralization. Furthermore, the article highlights a key research need, which is the assessment of the toxicity of transformation products and their validation under realistic conditions. Overall, this review provides insight into the potential and challenges of PAA-based AOPs for sustainable water treatment.

## 1. Introduction

In the face of increasing pollution of aquatic environments, water and wastewater treatment has become a key concern for both environmental and public health. Freshwater shortages are affecting an increasing number of regions worldwide, driven by climate change, rising demand from agriculture and industry, and inefficient resource management. It is estimated that in the coming years, more than half of the global population will face difficulties accessing clean water. Addressing this challenge requires not only reduced water consumption but also the implementation of effective and modern treatment technologies that can respond to emerging threats [1,2,3,4].

Traditional water and wastewater treatment systems, while effective in removing suspended solids, nutrients, and pathogens, often fall short in addressing trace organic pollutants that have only recently come into scientific and regulatory focus. These compounds, commonly referred to as contaminants of emerging concern (CECs), encompass a diverse group of substances, including pharmaceuticals, personal care products (PCPs), pesticides, industrial chemicals (ICBs), microplastics (MPs), and their metabolites [5]. Although they are typically present at low concentrations (ng/L to µg/L), CECs pose potential risks to human health and aquatic ecosystems due to their persistence and pseudo-persistence, bioaccumulation, and ability to exert toxicological effects even at trace levels. Moreover, the continuous discharge of these compounds into water bodies through municipal and industrial wastewater effluents contributes to long-term environmental exposure [6,7,8]. Plastic pollution is also a serious and alarming threat to ecosystems. Small particles, such as microplastics (less than 5 mm) and nanoplastics (typically less than 100 nm), pose a significant global problem due to their adverse environmental and public health impacts. Due to their small size, these particles are widely dispersed in terrestrial, freshwater, and marine environments, where they can be readily ingested by a diverse range of organisms, from plankton to higher trophic levels. Moreover, their presence in drinking water, food chains, and even atmospheric deposition highlights the urgency of addressing plastic contamination not only as an ecological hazard but also as a public health issue of global dimensions [9,10,11,12,13,14]. Consequently, pollution from both microplastics and CECs requires comprehensive research and coordinated environmental policy responses to limit their production, improve waste management systems, and develop advanced water treatment technologies capable of effectively removing these pollutants from aquatic environments [2,4,8]. The need to improve the removal efficiency of CECs has led to significant interest in advanced treatment strategies, among which advanced oxidation processes (AOPs) have emerged as particularly promising. AOPs are based on the in situ generation of highly reactive species, mainly hydroxyl radicals (HO•), which possess a high oxidation potential (2.8 V) and can non-selectively react with a broad spectrum of organic pollutants, ultimately leading to their mineralization or transformation into less harmful compounds. Various AOPs have been explored, including ozonation, UV/H_2_O_2_, Fenton and photo-Fenton processes, and combinations of oxidants and catalysts [15,16,17,18,19]. A review of the current literature concerning the application of AOPs reveals that the development and identification of novel reagents that combine high oxidative efficiency with environmental sustainability remains a subject of considerable research interest. Among the reagents recently proposed for AOPs, peroxyacetic acid (PAA) has attracted increasing attention due to its strong oxidative potential, relatively low environmental impact, and potential for in situ generation, which may further enhance its applicability in green chemistry frameworks.

In view of the above, the authors conducted a systematic literature review of PAA applications for the degradation of CECs. The review was conducted through comprehensive searches of the Science Direct, Scopus, and Google Scholar databases, encompassing publications from January 2020 to June 2025. The search strategy employed various combinations of the following keywords: “peracetic acid”, “contaminants of emerging concern”, “advanced oxidation processes”, and “microplastic”, to identify current research trends and knowledge gaps in this field.

Several review articles have already provided valuable syntheses of PAA-based AOPs, particularly regarding activation mechanisms, reactive species, catalytic systems, disinfection, operational parameters, and the degradation of selected organic pollutants [19,20,21,22,23]. The present review complements these works by providing an updated, pollutant-oriented synthesis that links PAA activation strategies with the degradation of major CEC classes and the oxidative modification of microplastics. Beyond summarizing removal efficiencies, this review critically discusses the distinction between apparent pollutant removal, transformation, and mineralization, as well as matrix effects, transformation product toxicity, secondary nanoplastic formation, and practical limitations relevant to real-water and wastewater treatment. This broader perspective is intended to support a more realistic evaluation of PAA-based AOPs beyond laboratory-scale degradation efficiency.

## 2. Characteristics of PAA

**Figure 1:**
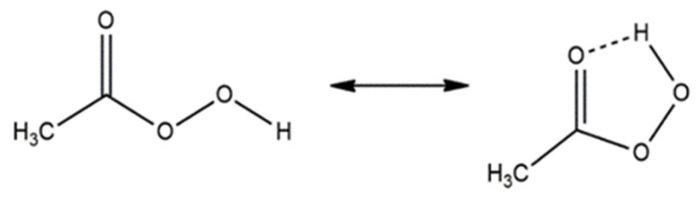
Structure of PAA.

PAA is an organic peroxyacid widely used in various applications as a disinfectant, sanitizer, bleaching agent, sterilizing agent, oxidizer, and polymerization catalyst [20]. Recently, its application as an oxidizing agent in water and wastewater treatment has attracted increasing attention. This interest results from its strong oxidizing properties, broad-spectrum antimicrobial activity, and environmentally benign decomposition products. PAA is commonly used in sectors such as the food industry, healthcare, and textiles, where it serves as an effective alternative to chlorine-based compounds [24,25,26]. Valued for its rapid action and for decomposing into acetic acid, oxygen, and water, making it environmentally friendly and safe [27,28]. The susceptibility of microorganisms to PAA generally follows the order bacteria > viruses > bacterial spores > protozoan cysts, and its effectiveness depends on both exposure time and concentration. [27,29,30]. Consequently, PAA is applied in municipal and industrial wastewater treatment and in healthcare disinfection, where it can also help reduce antibiotic-resistant bacteria and eliminate certain chlorine-tolerant clinical strains [31,32,33]. According to IUPAC nomenclature, PAA is known as ethaneperoxoic acid, but common names such as peroxyacetic acid, peracetic acid, acetyl peroxide, or peroxyethanoic acid are widely used [21]. PAA (CH_3_C(O)OOH) is classified as an organic peroxide, and its molecular structure is presented in Figure 1. PAA has a five-membered ring structure [34], which stabilizes its neutral form (PAA^0^), making it more prevalent than its anionic counterpart (PAA^−^) even in mildly alkaline aqueous environments. Furthermore, the neutral form (PAA^0^) exhibits a stronger oxidizing potential compared to the anionic form [19]. The chemical and physical properties are listed in Table 1.

PAA is a clear liquid with a smell very similar to vinegar. It dissolves well in water, ethyl alcohol, diethyl ether, sulfuric acid, acetic acid, and other organic solvents [35]. Commercially, PAA most often exists as a multi-component mixture containing PAA, acetic acid, hydrogen peroxide, and water (Equation (1)) [19,36].

(1) $${CH}_{3}COOH+H_{2}O_{2}↔{CH}_{3}COOOH+H_{2}O$$

PAA exhibits an oxidation potential (E_h_^0^) ranging from 1.0 to 1.96 V versus the standard hydrogen electrode (SHE) [36,37,38]. Which is higher than hydrogen peroxide (1.78 V), chlorine (1.48 V), chlorine dioxide (1.28 V), and ferrate (VI) (0.9–1.9 V) but lower than ozone (2.08 V) [19,24,36,39]. In comparison to acetic acid, PAA has a higher pK_a_ value (4.7 < 8.2), a lower boiling point (118 °C > 110 °C), a lower melting point (16.7 °C > 0.2 °C), and a smaller Henry’s law constant (7.36 × 10^4^ M·atm^−1^ > 4.68 × 10^2^ M·atm^−1^) [24,36,40]. A summary of oxidation potentials for common oxidants used in water treatment processes is presented in Table 2.

As an organic peroxide, PAA is thermodynamically unstable and may undergo decomposition or explosion upon heating or mechanical shock. The thermal stability of PAA is primarily determined by the dissociation energy of the peroxide O–O bond. PAA possesses a weaker peroxide bond (159 kJ·mol^−1^) compared to hydrogen peroxide (213 kJ·mol^−1^) [16,34,42]. The spontaneous decomposition of PAA proceeds via two mechanisms. The first involves the acid’s self-decomposition induced by protonation. This process consists of three steps: protonation, formation of an active intermediate, and generation of final products—two molecules of acetic acid and one molecule of oxygen (Equation (2)) [34,41]. The second mechanism, occurring within the pH range of 5.5–10.2, involves the nucleophilic attack of the peracetate anion on a PAA molecule, resulting in the formation of an active intermediate that decomposes into two acetate ions, molecular oxygen (O_2_), and a proton (H^+^) (Equation (3)) [34,41,42].

(2) $$2{CH}_{3}COOOH\to 2{CH}_{3}COOH+O_{2}$$

(3) $${CH}_{3}COOO^{-}+{CH}_{3}COOOH\to 2{CH}_{3}COO^{-}+O_{2}+H^{+}$$

## 3. PAA Oxidation Potential

PAA possesses a notably high redox potential, which underpins its strong oxidative capabilities [19,39,41,42]. Furthermore, structural analyses reveal that the peroxy bond length in PAA is slightly extended (1.443 Å) compared to H_2_O_2_ (1.427 Å) [34,41,42]. This relatively weak, elongated bond is more prone to cleavage, facilitating the generation of reactive radical species, such as hydroxyl and acetyl radicals (Equation (4)).

(4) $${CH}_{3}COOOH\to {CH}_{3}COO•+HO•$$

Despite its inherent thermodynamic instability, the spontaneous homolytic cleavage of the O–O bond in PAA occurs at a very slow rate, with a rate constant of k = 6 × 10^−12^ s^−1^ [42,43,44]. This low rate limits its practical efficiency unless external activators are introduced. These activators enhance the cleavage kinetics of the peroxy linkage, thereby increasing the yield of reactive oxygen species necessary for effective oxidation processes. In AOPs based on PAA, the generation of reactive radical species is a crucial step for purifying water and wastewater and for oxidizing organic micropollutants (OMPs) present in these matrices. PAA can be activated via radical or non-radical pathways. Radical mechanisms typically lead to the formation of highly reactive species such as HO• and R-O•. Non-radical pathways, though less well understood, may involve the formation of high-valent metal-PAA complexes or singlet oxygen (^1^O_2_) [19,23,42,45]. Various activation methods, including UV or light irradiation, ultrasonic treatment, or the use of catalysts such as metal ions or activated carbon, can initiate this process. Activation approaches differ fundamentally in (i) dominant reactive species generated, (ii) selectivity toward pollutant classes, (iii) sensitivity to water matrix components, and (iv) operational feasibility. From this perspective, PAA activation methods can be broadly categorized into three mechanistic regimes:

(1) Photochemical, sonochemical, and thermochemical systems dominated by hydroxyl radical formation,
(2) Metal-mediated systems governed by organic radicals and high-valent metal species,
(3) Carbon-based systems enabling electron-transfer-driven non-radical pathways.

These mechanistic differences determine not only oxidation efficiency but also robustness toward natural organic matter (NOM), halides, and carbonate species, which are critical under realistic water treatment conditions.

### 3.1. Physical Activations of PAA

#### 3.1.1. UV Irradiation

Ultraviolet (UV) irradiation is one of the earliest and most extensively investigated methods for PAA activation [23,42]. The UV energy efficiently induces homolytic cleavage of the peroxy bond in PAA, forming acetyloxy radical (CH_3_COO•) and hydroxyl radical (HO•) (Equation (4)). This stage plays a crucial role in energy emission and directly affects the degradation rate of pollutants. Additionally, the PAA solution contains a small amount of H_2_O_2_, which can also be activated by energy radiation, leading to the formation of HO• that participates in subsequent reactions (Equation (5)) [19,41,42,46].

(5) $$H_{2}O_{2}\to 2HO•$$

Thereafter, CH_3_COO• rapidly dissociates to methyl radical (CH_3_•), CO_2_, and CH_3_• may combine with oxygen to produce a weak CH_3_OO• radicals (Equations (9) and (10)). Meanwhile, HO• may attack the PAA molecule (Equations (6)–(8)). CH_3_COO• may also react with PAA to produce acetylperoxyl radical (CH_3_COOO•) (Equation (11)) [19,42,47].

(6) $${CH}_{3}COOOH+HO•\to {CH}_{3}COOO•+H_{2}O$$

(7) $${CH}_{3}COOOH+HO•\to {CH}_{3}CO•+O_{2}+H_{2}O$$

(8) $${CH}_{3}COOOH+HO•\to {CH}_{3}COOH+HOO•$$

(9) $${CH}_{3}COO•\to {CH}_{3}•+{CO}_{2}$$

(10) $${CH}_{3}•+O_{2}\to {CH}_{3}COO•$$

(11) $${CH}_{3}COOOH+{CH}_{3}COO•\to {CH}_{3}COOO•+{CH}_{3}COOH$$

PAA exhibits higher quantum yields (Φ = 0.88–2.09) than H_2_O_2_ (Φ = 0.5) at 254 nm, indicating its superior ability to generate reactive radicals. UV/PAA systems have demonstrated high efficiency in degrading pharmaceuticals and other pollutants, often outperforming UV/H_2_O_2_ systems [23,47]. However, its performance is strongly dependent on light penetration and is therefore limited in turbid or NOM-rich matrices. The predominance of hydroxyl radicals makes this pathway highly non-selective, enhancing broad-spectrum degradation but also increasing radical scavenging by bicarbonates, chlorides, and dissolved organic matter. Consequently, while UV activation is advantageous for low-strength and well-controlled waters (e.g., tertiary effluents), its efficiency may decrease significantly in complex matrices where radical quenching dominates. In contrast to metal-mediated activation, UV systems typically lack catalytic amplification mechanisms, which explains their lower performance in several studies despite favorable intrinsic radical yields.

#### 3.1.2. Ultrasound Irradiation (Sonochemistry)

Sonochemistry is based on the application of ultrasound, which induces acoustic cavitation in liquids, thereby forming chemically active species. This technology has found wide application in the degradation of pollutants, such as chlorinated organic compounds [48,49], as well as in the synthesis and modification of materials. Due to the absence of additional chemical reagents, sonochemistry is considered a green, environmentally friendly method applied in various fields, including organic chemistry, biomass valorization, electrochemistry, catalysis, environmental remediation, and polymer chemistry [49,50]. Over the past years, ultrasound has emerged as an effective AOP for wastewater treatment, primarily due to its ability to generate HO• in aqueous media, which subsequently drives the oxidation and degradation of organic pollutants [51]. The fundamental reactions occurring during sonication in aqueous dispersion, which can be regarded as initiators of a series of radical reactions, are represented by Equations (12)–(15) [51].

(12) $${CH}_{3}COOOH\to {CH}_{3}COO•+HO•$$

(13) $${CH}_{3}COOOH\to {CH}_{3}CO•+HOO•$$

(14) $$H_{2}O\to HO•+H•$$

(15) $$H_{2}O_{2}\to 2HO•$$

Since then, ultrasound has been investigated for wastewater treatment involving a wide spectrum of contaminants, including aromatic and chlorinated aliphatic compounds, explosives, herbicides, pesticides, organic dyes, gaseous pollutants of both organic and inorganic origin, organic sulfur compounds, oxygenates, alcohols, pharmaceuticals, PCPs, as well as waterborne pathogens and bacteria [52].

#### 3.1.3. Thermal Activation

Thermally induced homolytic cleavage of the O–O bond in the PAA molecule in aqueous solution is feasible, and the reactive radicals generated in this process (Equation (12)) exhibit significant potential for the removal of micropollutants [53]. Moreover, most chemical oxidation processes are generally temperature-dependent. Therefore, a detailed understanding of the thermal decomposition mechanism of PAA is crucial for its application in water and wastewater treatment [20,53].

### 3.2. Chemical Activations of PAA

#### 3.2.1. Transition Metal Ions

Metal-based catalysts are widely employed to activate oxidants by generating reactive species. Both homogeneous (dissolved transition metal ions) and heterogeneous (metal oxides, sulfides, and composites) catalysis approaches have been explored.

##### Homogeneous Metal Catalysis

The current transition metals, such as Fe^2+^, Cu^+^, Co^2+^, Ru^3+^, and Mn^2+^, can activate PAA through single-electron transfer (SET), both gaining and losing electrons, resulting in a cyclic activation mechanism (Equations (16) and (17)) [19,23,54]:

(16) *X*^*n*+^ + *CH*_3_*C*(*O*)*OOH* → *X*^(*n*+1)+^ + *CH*_3_*C*(*O*)*O*• + *OH*^−^

(17) *X*^(*n*+1)+^ + *CH*_3_*C*(*O*)*OOH* → *X*^*n*+^ + *CH*_3_*C*(*O*)*OO*• + *H*^+^

In the case of cobalt, Co^2+^ oxidizes to Co^3+^ while generating CH_3_COO•, and is subsequently reduced back by PAA, producing CH_3_COOO•, thus forming a redox cycle (Equations (18) and (19)) [23,54,55,56].

(18) $${{Co}^{2+}+CH}_{3}COOOH\to {CH}_{3}COO•+{Co}^{3+}+{OH}^{-}$$

(19) $${{Co}^{3+}+CH}_{3}COOOH\to {CH}_{3}COOO•+{Co}^{2+}+H^{+}$$

Therefore, during PAA consumption, cobalt in both oxidation states, Co^2+^ and Co^3+^, undergoes continuous regeneration. Put differently, both cobalt species participate in PAA decomposition, regardless of whether the process is initiated by Co^2+^ or Co^3+^, provided that the reactions described by Equations (18) and (19) proceed at similar rates. Mn^2+^ can also serve as a catalyst, facilitating the cleavage of O-O bonds within the PAA molecule [57]. In systems containing Mn^2+^ ions, the overall mechanism is most likely reactions with Co^2+^ [58]. Iron catalysis involves complex mechanisms and can produce R-O•, CH_3_C(O)O^−^, Feᴵⱽ, O_2_^+^, HO•, and CH_3_COOH. Homogeneous catalysts are highly efficient but pose challenges in recovery and reuse, as well as potential environmental risks from metal-ion leaching. Beyond efficiency, metal-mediated activation introduces a fundamentally different oxidation regime compared to UV systems. Cobalt-, iron-, and manganese-based catalysts predominantly generate organic radicals (e.g., CH_3_COOO•), which exhibit higher selectivity. This selectivity explains the usually higher degradation efficiencies of Co- and Fe-based systems compared to purely photolytic activation. However, this advantage is accompanied by important limitations, including sensitivity to pH (especially for Fe systems), secondary pollution by metals, and dependence on redox cycling efficiency. In real-water matrices, NOM may inhibit catalytic turnover by complexation of metal ions, while halides may redirect oxidation toward halogenated by-products. Thus, metal activation offers superior kinetics but reduced environmental compatibility compared to light-based systems.

The potential reactions taking place in the presence of Fe^2+^ ions are outlined below (Equations (20)–(25)) [19,59].

(20) $${{Fe}^{2+}+CH}_{3}COOOH\to {CH}_{3}COO•+{Fe}^{3+}+{OH}^{-}$$

(21) $${{Fe}^{3+}+CH}_{3}COOOH\to {CH}_{3}COOO•+{Fe}^{2+}+H^{+}$$

(22) $${{Fe}^{2+}+CH}_{3}COOOH\to {CH}_{3}COO^{-}+{Fe}^{3+}+HO•$$

(23) $${{Fe}^{2+}+CH}_{3}COOOH\to {CH}_{3}COOH+{Fe}^{IV}O^{2+}$$

(24) $${{2Fe}^{IV}O^{2+}+CH}_{3}COOOH\to {CH}_{3}COO^{-}+2{Fe}^{3+}+{OH}^{-}+O_{2}$$

(25) $${Fe}^{IV}O^{2+}+H_{2}O\to HO•+{Fe}^{3+}+{OH}^{-}$$

Hydrogen peroxide, which exists in equilibrium with PAA, also reacts with Fe^2+^ ions [60]. The equations (Equations (26)–(29)) illustrate the course of these reactions, known as the Fenton reaction.

(26) $${Fe}^{2+}+H_{2}O_{2}\to HO•+{Fe}^{3+}+{OH}^{-}$$

(27) $${Fe}^{2+}+H_{2}O_{2}\to {Fe}^{IV}O^{2+}+H_{2}O$$

(28) $${Fe}^{3+}+H_{2}O_{2}\to HO_{2}•+{Fe}^{2+}+H^{+}$$

(29) $${Fe}^{IV}O^{2+}+H_{2}O\to HO_{2}•+{Fe}^{3+}+{OH}^{-}$$

##### Heterogeneous Metal Catalysis

Heterogeneous catalysts, such as metal oxides (e.g., Co_3_O_4_, MnO_2_, LaCoO_3_), sulfides (e.g., MoS_2_, FeS), and composites (e.g., CoFe_2_O_4_, Fe^2+^-zeolites, Co@MXenes)**,** enable efficient PAA activation while being easily separable and usable over a broad pH range. These catalysts operate via electron-transfer mechanisms similar to those of their homogeneous counterparts. Non-metallic elements such as S and N in the catalysts can serve as electron donors, further enhancing catalytic performance. For instance, Fe^2+^ sites in FeS_2_ serve as active centers, while sulfides aid Fe^3+^ reduction, sustaining PAA activation. Cobalt-based catalysts have shown the highest activity, primarily due to the Co^2+^/Co^3+^ redox cycling. Iron-based catalysts, though less studied, also exhibit notable performance, especially when supported on zeolites [23,60]. Importantly, heterogeneous systems extend the operational window of PAA activation by enabling near-neutral pH performance and improved resistance to radical scavengers. Their surface-mediated electron-transfer pathways allow partial bypassing of free-radical quenching, a key limitation in homogeneous systems. Nevertheless, the high activity of cobalt-based materials raises concerns regarding long-term stability and environmental safety, highlighting a trade-off between catalytic efficiency and sustainability that must be considered for full-scale applications.

Among transition metal activators, cobalt-based systems consistently demonstrate the highest PAA activation efficiency. This is primarily due to the favorable Co(II)/Co(III) redox cycle, which enables the rapid and continuous generation of reactive organic radicals such as CH_3_C(O)O• and CH_3_C(O)OO• under near-neutral pH conditions. Compared to iron-based systems, cobalt activation is less dependent on acidic conditions and often exhibits faster kinetics and higher stability in complex matrices. In contrast, iron(II)-based activation is often limited by narrow pH ranges and the precipitation of iron hydroxides, while manganese (Mn)-based systems typically exhibit slower activation kinetics. However, despite their high catalytic efficiency, cobalt-based systems raise significant practical concerns. In particular, partial decomposition of Co-based species under oxidizing conditions can lead to measurable cobalt leaching, especially in heterogeneous systems. This not only shortens the catalyst’s lifetime but also introduces the risk of secondary metal contamination. Furthermore, the relatively high cost of cobalt precursors and the need for controlled synthesis of stable catalyst structures may limit their economic viability on a large scale. Long-term operation is further complicated by potential surface deactivation, structural changes, and loss of active sites during repeated cycles. Therefore, despite excellent laboratory results, the ecological and operational sustainability of cobalt-based systems remains a key challenge for practical implementation.

#### 3.2.2. Carbon-Based Catalysts

The diversity of molecular hybridization and surface chemistry of carbocatalysts creates favorable adsorption sites for both PAA and pollutants, thereby facilitating oxidation processes. Carbon materials can generally be classified into two main groups: pristine carbons and chemically or structurally modified carbons. The first category includes nanocarbon materials and bulk carbons, whereas the second group comprises chemically doped carbons, carbon-metal composites, and carbon-based single-atom catalysts. Due to their high flexibility in chemical surface properties, large specific surface area, and porosity, carbonaceous materials represent an advanced platform for designing and controlling potential active sites, such as structural defects, surface functional groups, non-metal heteroatom dopants, anchored single-metal atoms, and various forms of carbon hybridization. The targeted engineering of carbon materials to impart optimized physicochemical and electronic properties can stimulate PAA activation, leading to the oxidation of organic pollutants via either radical or non-radical pathways [23,45,60]. The activation of PAA by carbonaceous materials primarily proceeds via electron transfer mechanisms. Various active sites present on the carbon surface play key roles in this process. In addition to homolytic cleavage of the peroxy bond, producing HO• and CH_3_COO• radicals, ^1^O_2_ can also be generated from carbon materials activating PAA. The mechanism of PAA activation by carbon-based catalysts may differ from that of metal-based catalysts and may proceed via non-radical pathways involving direct electron transfer or singlet oxygen formation. This feature provides a critical advantage in conditions where radical scavenging limits the effectiveness of conventional AOPs. Carbonaceous catalysts often exhibit slightly slower kinetics but greater stability across varying water chemistries. Their performance is less affected by carbonate and chloride ions, making them promising candidates for treating complex wastewaters. However, the absence of strong radical flux may reduce their effectiveness against highly recalcitrant compounds, suggesting a complementary rather than universal role as activation platforms. Carbon materials with high π-electron content can facilitate the formation of PAA, which subsequently promotes a nonradical electron transfer mechanism with pollutants. This implies that the activation mechanism of PAA by carbon catalysts can be tuned between radical and nonradical pathways by controlling the sp^3^/sp^2^ carbon ratio [23,45,60].

### 3.3. Comparative Assessment of PAA Activation Strategies

To facilitate comparison of the activation strategies discussed above, the main practical trade-offs between physical and chemical PAA activation methods are summarized in Table 3. Physical methods are attractive because they avoid catalyst addition and reduce the risk of secondary contamination, but they are limited by the need for continuous energy input and low selectivity in complex matrices. Chemical methods may provide faster kinetics, catalytic cycling, and improved selectivity or matrix tolerance, but they introduce additional challenges related to pH dependence, catalyst recovery, stability, and possible metal leaching.

## 4. PAA Oxidation of CECs

In recent decades, there has been a growing interest in so-called CECs. This term refers to a broad group of chemical compounds that occur in the aquatic environment at very low concentrations (from ng/L to µg/L), yet can still exert significant toxicological effects on aquatic organisms and human health. These substances, although often not sufficiently regulated by law or lacking established environmental standards, are causing increasing concern due to their persistence, bioaccumulation potential, and endocrine-disrupting properties [5,61,62]. Conventional wastewater treatment methods, applied in most municipal wastewater treatment plants, primarily involve mechanical, biological, and chemical processes that are not fully effective at eliminating CECs [1,5]. The removal of micropollutants in classical systems relies mainly on sorption to activated sludge and partial biodegradation under aerobic and anaerobic conditions. Studies indicate that the reduction efficiency of many pharmaceuticals, pesticides, and endocrine-disrupting compounds (EDCs) in conventional wastewater treatment plants typically ranges from a few to several tens of percent, depending on the compounds’ chemical properties and the technological conditions [4]. Chemical precipitation and coagulation processes, commonly used in water and wastewater treatment, also demonstrate limited effectiveness against CECs. Therefore, conventional technologies are currently considered insufficient and require the development of new, more effective methods for eliminating these pollutants. AOPs are gaining increasing importance because they generate reactive radicals that effectively degrade persistent micropollutants into simpler compounds. The most widely applied AOPs in wastewater treatment include ozonation, UV/H_2_O_2_ processes, Fenton and photo-Fenton reactions, photocatalysis using TiO_2_, and emerging electrochemical processes [18].

Compared with conventional AOPs, PAA-based systems should be considered as complementary rather than universally superior technologies. UV/H_2_O_2_ is well established and effective for broad-spectrum oxidation, but its HO•-dominated chemistry is highly susceptible to scavenging by NOM, bicarbonates, carbonates, and other background constituents in complex matrices. Fenton and photo-Fenton processes provide rapid degradation kinetics, yet they are often limited by acidic pH requirements, iron sludge formation, and post-treatment needs. Ozonation is mature and effective for many electron-rich contaminants, but it may involve high energy demand, incomplete mineralization, and by-product formation such as bromate in bromide-containing waters. PAA-based AOPs can also generate HO•; however, depending on the activation pathway, organic radicals such as CH_3_C(O)O• and CH_3_C(O)OO•, as well as non-radical routes, may substantially contribute to contaminant degradation. These pathways may improve selectivity toward electron-rich compounds and reduce, but not eliminate, the negative impact of radical scavenging in complex matrices. Nevertheless, PAA-based AOPs are still less mature than conventional AOPs, and their implementation remains limited by catalyst stability, management of residual PAA/acetic acid, transformation product toxicity, and cost. Therefore, the selection of PAA-based or conventional AOPs should depend on target pollutants, matrix composition, treatment objectives, and full-scale feasibility. The growing demand for more efficient oxidants than hydrogen peroxide or ozone has stimulated interest in PAA in recent years. CECs comprise a wide range of chemical compounds that differ significantly in their structure, physicochemical properties, and applications. The following sections describe the main CEC categories: microplastics, pharmaceuticals, personal care products, pesticides, and industrial chemicals and by-products.

### 4.1. PAA Oxidation of Microplastic

MPs are commonly defined as plastic particles with a size ranging from 1 µm to 5 mm. These particles are categorized into two main types: primary and secondary MPs. Primary MPs are manufactured at microscopic sizes for specific uses, such as microbeads in PCPs or industrial abrasives. In contrast, secondary MPs result from the breakdown of larger plastic items through physical, chemical, or biological processes, such as UV radiation or mechanical abrasion [14]. In contemporary environmental matrices, the most frequently detected MPs are commodity polymers polyethylene (PE), polypropylene (PP), polystyrene (PS), polyethylene terephthalate (PET), and polyvinyl chloride (PVC). They are ubiquitously detected in freshwater systems, marine environments, and in atmospheric deposition, such as urban dust and aerosols [63,64]. Their ability to adsorb persistent organic pollutants (POPs), heavy metals, and pathogens further exacerbates their ecological risk.

For oxidative degradation of MPs, most often hydroxyl radical routes (UV/H_2_O_2_, Fenton and photo-Fenton) and sulfate radical routes using persulfate (PDS) or peroxymonosulfate (PMS) are employed, alongside photocatalysis and ozone-based systems [65,66]. A review of the literature indicates that only a limited number of studies have explored the oxidative potential of PAA for the degradation of the MPs most frequently encountered in the environment [67,68].

In the study by Kong et al., the oxidation of PE, PS, and PET in the UV/PAA system was examined [67]. The photolysis of PAA leads to the generation of CH_3_COO• and HO•, followed by surface degradation of the polymers and modification of their properties. The UV/PAA system showed a pronounced impact on PS, whereas the modifications observed in MPs with simple hydrocarbon chains (PE, PP) were more moderate. Table 4 summarizes the examined parameters, obtained results, and observed changes in oxidation of the studied MPs in the UV/PAA system.

XPS spectroscopy and C/O ratio indicate new oxygen-containing hydrophilic groups formed on the surface of MPs after UV/PAA treatment. In PP, carbonyl (C=O) and ester groups were formed; in PE, additional hydroxyl groups (-OH) were formed; and in PS, a decrease in aromatic band intensity was also observed. However, for PE after UV/PAA treatment, the C/O ratio increased from 31.6 to 51.4, whereas for PP (from 111.4 to 28.1) and PS (from 65.2 to 7.0), it decreased significantly. The change in the C/O ratio after UV/PAA treatment indicates different mechanisms of polymer degradation in PE, the loss of oxygen or the formation of carbon products dominates (an increase in the value of in C/O ratio), while in PP and PS, intense oxidation introduces more oxygen into the structure (a decrease value of C/O ratio). The changes included the introduction of polar groups and the enhancement of sorption properties, which are important for assessing their subsequent fate in the aquatic environment.

Zaritskii [68] investigated the chemical resistance and mechanical changes of PE, PVC, and fluoroplast-4 (PTFE) in an aqueous PAA/H_2_O_2_ system (CH_3_COOOH 4%, H_2_O_2_ 5%, H_2_O 91%) under ambient conditions. Very high resistance of PTFE was observed, showing no significant changes after 1000 hs and retaining its mechanical properties. PE and PVC underwent minor modifications: initial swelling was observed (K ≈ 0.09% for PE, 0.11% for PVC at 500–600 h), followed by gradual dissolution, with final values of 0.01% (PE) and 0.014% (PVC) after 1000 h of oxidation (Table 5).

PE and PVC exhibited limited chemical modification, highlighting the role of polymer composition in chemical stability under oxidative conditions. PVC was found to be more effective at promoting PAA dissociation than PE or PTFE, suggesting chemical interactions at the interface. Modifications of MPs by PAA/H_2_O_2_ system. Available studies on MPs primarily report surface oxidation, formation of oxygen-containing functional groups, and changes in crystallinity and hydrophobicity rather than complete polymer degradation [41,69]. Available evidence indicates that PAA-based systems primarily induce oxidative aging of the polymer surface rather than extensive backbone cleavage or mineralization. This behavior is consistent with the relatively selective reactivity of PAA, which preferentially attacks sensitive surface defects and functional groups rather than promoting nonselective cleavage of highly stable C–C bonds in polymers such as PE, PP, and PS. Advanced PAA-based oxidation processes are typically characterized by lower mineralization efficiency than hydroxyl-dominated systems, unless catalytic activation is employed, and oxidation is often limited to the transformation of surface functionalities rather than complete carbon mineralization [41]. Consequently, oxidation primarily introduces carbonyl, hydroxyl, and carboxyl groups, increasing surface polarity and oxidative embrittlement. Similar oxidative aging phenomena, including surface cracking, increased oxygenation, hydrophobicity changes, and progressive embrittlement, have been widely reported for microplastics aged under environmental conditions and are considered precursors to fragmentation into smaller particles [69]. Such partial oxidation can promote cracking, embrittlement, and particle size reduction, potentially leading to the formation of secondary nanoplastics rather than complete detoxification. Therefore, an apparent decrease in visible microplastic particles should not automatically be interpreted as successful degradation. In some cases, oxidative weathering may simply redistribute contaminants from microplastics to nanoplastics, which may be even more hazardous to the environment due to their greater mobility, bioavailability, and potential for cellular uptake. Recent systematic analyses of microplastics aged under AOP conditions indicate that fragmentation and the formation of secondary nanoplastics can significantly increase environmental risk, despite the apparent removal of larger particles [70]. To distinguish fragmentation from true degradation, future studies should combine particle characterization with mineralization and molecular weight analysis. Recommended methods include measuring total organic carbon (TOC) and dissolved organic carbon (DOC), monitoring CO_2_ evolution, and performing size-exclusion chromatography (SEC/GPC) to verify true polymer chain scission and mineralization. In parallel, nanoparticle tracking analysis (NTA), dynamic light scattering (DLS), and high-resolution imaging techniques such as scanning electron microscopy (SEM), transmission electron microscopy (TEM), or atomic force microscopy (AFM) should be used to quantify the formation of secondary nanoplastics and distinguish surface erosion from bulk degradation. Pyrolysis-GC/MS can additionally aid in identifying oxidation products, assessing weathering-induced chemical changes, and establishing polymer mass balance, and is currently considered one of the most robust analytical approaches for MP/NP characterization [71].

Without such integrated analyses, reductions in microplastic abundance may be overestimated and misinterpreted as effective degradation. Furthermore, the ecotoxicological assessment of oxidation byproducts and secondary nanoplastics remains largely unexplored. This represents a significant research gap, especially since surface oxidation can significantly alter the sorption of pollutants and the fate of microplastics in the environment.

### 4.2. PAA Oxidation of Other CECs

The literature review indicates that AOPs methods based on PAA utilization were used for pharmaceuticals, PCPs, pesticides, and ICBs removal from water. The most commonly used PAA activators include UV irradiation, transition metals (Co, Fe, Mn), metal complexes, carbon catalysts, heterogeneous oxides and composites, and halides.

#### 4.2.1. Pharmaceuticals

Pharmaceutical compounds encompass a wide spectrum of active substances, including analgesics, anti-inflammatory drugs, antibiotics, antiepileptic drugs, antidepressants, β-blockers, and synthetic hormones [5,72]. Their persistent release into the environment comes primarily from human and animal feces, hospital and domestic wastewater, and incomplete removal in conventional wastewater treatment plants (WWTPs). Concentrations in surface waters typically range from ng/L to mg/L, reflecting their pseudopersistence, a balance between continuous emissions and limited degradation [73].

Pharmaceuticals are often polar and resistant to biodegradation, allowing them to bypass biological treatment processes. Antibiotics and their metabolites are of particular concern because they contribute to the selection of antibiotic-resistant bacteria and genes (ARB/ARG), posing a serious global health threat [5]. Furthermore, endocrine disrupting drugs such as ethinylestradiol (EE2) and diclofenac (DCF) disrupt the hormonal balance of aquatic organisms, causing feminization, reproductive dysfunction, and population-level effects. Chronic exposure of the aquatic environment to that level of concentration has been shown to disrupt enzymatic systems and oxidative metabolism, and accumulation in sediments and biota facilitates trophic transfer [72]. Their presence in the environment is becoming an increasingly serious problem [73]. Their potential impact on ecosystems and human health is currently being intensively studied. Research highlights the importance of analyzing the presence and assessing the associated ecological risk. Reducing the amount of micropollutants released into the environment is also crucial. To better understand the scale of the problem and the potential of PAA, this section discusses studies evaluating its effectiveness in removing PAA [5,73]. Table 6 summarizes numerous recent scientific publications that use various activators to remove pharmaceuticals using PAA.

The degradation of pharmaceuticals by PAA activation was studied using a diverse set of activators, ranging from homogeneous metal ions and radical promoters to heterogeneous catalysts and photo-assisted processes. The collected data indicate a strong dependence of the removal efficiency on both the choice of activator and the reaction conditions. Activated PAA showed high degradation efficiency for a group of pharmaceuticals such as antibiotics (AMX, SMX, TMP, SDZ, TC, MOX, CTC, OTC, CFX, AMP, and SMZ), antifungals (FCL), hormones (EE2, E1), non-steroidal anti-inflammatory drugs (NSAIDs) (NPX, IBU, DCF, ACT), cardiovascular (PPL, BZF), antiepileptics (CBZ) (Table 5) under different conditions and different times. Works by Kim et al. [56] and Liu et al. [85] present homogeneous Co^2+^ activation (100 µM PAA combined with 10 µM Co^2+^), which was frequently used and demonstrated consistent efficacy for different pharmaceuticals, achieving 87.2% removal of SMX, 93.4% of NPX, and 61.8% of CBZ within 30 min at neutral pH. Co^2+^ activates PAA, generating CH_3_COO• and CH_3_COOO• radicals with minimal hydroxyl HO• radical formation. In contrast, in studies by Wang et al. [76] and Zhou et al. [84], heterogeneous spinel ferrites such as CoFe_2_O_4_ showed even faster and more complete activity; specifically, at doses ranging from 0.1 to 0.5 g/L with 200–800 µM PAA, degradation of model drugs such as DCF and SMX reached efficiencies reaching 97–100% within 10 min. Xue et al. [89] used magnetic biochar-ferrospinel AFe_2_O_4_ (A = Co^2+^, Mn^2+^, Cu^2+^) nanocomposites in their research. Optimal conditions for BC-CoFe_2_O_4_/PAA were 0.8 mM PAA, 0.3 g/L catalyst, and a pH close to neutral, which resulted in 100% CBZ removal. The dominant active species are CH_3_COOO• radicals, responsible for CBZ degradation in the BC-CoFe_2_O_4_/PAA system. Although BC-MnFe_2_O_4_/PAA and BC-CuFe_2_O_4_/PAA systems achieved only 7% CBZ removal in the same conditions. Other transition metal-based nanomaterials, including, zero-valent cobalt (ZVCo), and cobalt carbon nitride (Co-CN), also provided near-quantitative removal, highlighting the catalytic superiority of cobalt-based systems [75,84]. Approximately 99% SMX could be removed by ZVCo/PAA under neutral conditions in 5 min. SMX degradation followed a pseudo-first-order (PFO) kinetic model organic radicals CH_3_C(O)O• and CH_3_C(O)OO• were the dominant reactive species for SMX degradation. The Co-CN/PAA system achieved 98.5% SMX removal in 360 s at pH 6.5, with 0.1 mM PAA and 50 mg/L Co-CN. SMX degradation in the Co-CN/PAA system followed a PFO kinetic model. PAA activation occurs due to surface Co^2+^ on Co-CN, which react with PAA to generate organic radicals CH_3_C(O)O• and CH_3_C(O)OO•. This research article [93] details the development and mechanisms of an AOP using nanoparticle zero-valent iron (nZVI)-activated PAA for the efficient removal of TC and homogeneous Fe^2+^/PAA was also investigated for CTC, OTC, CFX, and AMP. The study highlights that Fe^2+^ complexation with TC significantly accelerates the decomposition of PAA and the generation of CH_3_C(O)OO• radicals, which are the main active species responsible for degradation. The nZVI/PAA and Fe^2+^/PAA process exhibited superior TC, CTC, OTC, CFX, AMP abatement efficiency (>95%) within 5–30 min. The same author presented the results of DCF degradation studies using the ZVC/PAA system [94]. Optimal conditions was 100 µM PAA, 0.5 g/L ZVC, pH 2.0, and DCF was completely removed within 40 min by the ZVC/PAA system. DCF degradation followed a PFO kinetic model, PAA was effectively catalyzed by ZVC to produce HO•, CH_3_COO•, and CH_3_COOO•. Metal–organic support frameworks (MOFs), such as bimetallic Fe/Co composites, represented another class of efficient activators and was used for degradation of SMX. Fang et al. [81] in this study achieving 98.1% BZF degradation was reached in 60 min using the MOF-(Fe1,Co1)/PAA system. For other tested pollutants, high degradation efficiencies were observed: IBU (74.9%), DCF (97.1%), CBZ (97.1%), MOX (99.5%), SMX (99.7%), and PPL (99.8%) at relatively low catalyst loadings (10 mg/L MOF with 25 mg/L PAA). In this case a synergistic interaction between Fe and Co on the MOF-(Fe,Co) surface enhanced PAA activation, leading to the generation of R–O•, Fe(IV)/Co(IV), and O^•−2^ radicals. Furthermore, Zhou et al. [96] developed and achieved 100% SMX removal after 12 min under Mn_3_O_4_/PAA system 1 mM PAA, 50 mg/L catalyst. CH_3_C(O)O•, CH_3_C(O)OO•, and HO• were identified as key radicals, with ^1^O_2_ potentially forming through PAA self-decomposition. Wang et al. [82] presented iron-based materials, such as Fe^2+^-zeolite, with conditions 400 μmol/L PAA, 0.8 g/L Fe^2+^-zeolite completely removing SMX was observed within 50 min at mild pH 7.0. SMX degradation in Fe^2+^-zeolite/PAA followed a PFO kinetic model, HO• was proved to be mainly responsible for oxidation. In addition, Wang et al. [87] presented interesting research using the Fe^3+^/MoS_2_/PAA system. A key innovation is the use of molybdenum disulfide (MoS_2_) as a catalyst to support the activation of PAA in the presence of Fe^3+^. The researchers found that the addition of MoS_2_ significantly accelerates the degradation of SMX, generating a variety of reactive oxygen species, such as Fe(IV), HO• and CH_3_COOO•. A total of 96.8% SMX degradation was achieved in the Fe^3+^/MoS_2_/PAA process after 10 min. This study not only establishes the optimal conditions for this process but also demonstrates the feasibility of recycling MoS_2_ in a flow reactor, suggesting its practical application in water purification. Nonmetallic catalysts, such as thermally modified activated carbon (AC600), also showed promising potential, although they required removal efficiency 99.4% SMX in 150 min [86]. Electron-transfer processes (ETPs) and organic radicals, including CH_3_C(O)O• and CH_3_COOO• were responsible for SMX degradation. In addition to solid catalysts, photoactivation methods attracted considerable interest from the authors. Santiago-Espiñeira et al. [74], Al. Umairi et al. [78] and Hollman et al. [81] used UV-C/PAA and UVA/PAA systems in their works, which induced the degradation of SMX and AMX, although their use alone was often less efficient, ranging from 34% to 78%. However, hybrid systems combining UV with metal or anion activators, such as UV/PAA/NO_2_^−^ [90] or PAA/UVC-LED/Fe^2+^ [91], were significantly more efficient, achieving >95% degradation of SDZ and ACT, respectively, in only 10–30 min. The study by Du et al. [79] used bromide-assisted activation (PAA/Br^−^), which was another noteworthy pathway, enabling the removal of 81% of SMX, 48.7% of EE2, and 31.3% of NPX within 1 h, reaction conditions: 0.2 mM PAA, 0.2 mM Br^−^, pH 7.1. Also, pharmaceuticals such as SMX, DCF, and SMZ can be removed using LaCoO3/PAA [73], PBS/PAA [93], and ABC/PAA [95], respectively. Their removal efficiency in these systems ranges from 72.8 to 95.7% within 50–100 min. The HO• radical, CH_3_COOO•, and CH_3_COO• were identified as the dominant reactive species responsible for the degradation of these compounds. The literature data presented indicate that transition metal-mediated activation, particularly in systems based on Co^2+^, CoFe_2_O_4_, and Fe, provides the highest removal efficiency of pharmaceutical compounds, often exceeding 90% in a short reaction time at near-neutral pH conditions. This higher efficiency is due to the efficient activation of PAA and the dominant formation of reactive organic radicals, particularly CH_3_COOO• and CH_3_COO•, which are highly effective for electron-rich pharmaceutical structures. In contrast, UV/PAA systems typically exhibit lower degradation efficiency and require longer reaction times unless combined with additional activators such as Fe^2+^, NO_2_^−^, or halides. However, the practical application of metal-mediated systems remains limited by catalyst stability, metal leaching, sensitivity to natural organic matter (NOM), and possible inhibition by bicarbonates and chlorides present in real wastewater matrices. Moreover, high contaminant removal does not necessarily correspond to complete mineralization, and the formation and toxicity of transformation products are still under-studied. Therefore, future research should focus not only on degradation efficiency but also on catalyst recyclability, matrix effects, byproduct toxicity, and validation under realistic treatment conditions.

#### 4.2.2. Personal Care Products (PCPs)

Compared to pharmaceuticals, research on the degradation of PCPs via PAA activation is more limited but offers valuable insights into the approach’s feasibility. PCPs encompass a diverse array of chemicals used in cosmetics, perfumes, sunscreens, disinfectants, and hygiene products. Common substances such as triclosan (TCS), triclocarban (TCC), parabens, benzophenones, and synthetic musks are chemically stable, lipophilic, and susceptible to sorption by sediment or organic matter [5,62,73]. Their widespread use in households and constant discharge into wastewater make them ubiquitous environmental pollutants. PCPs often act as EDCs, imitating or blocking natural hormones in humans and animals [74,97]. For example, TCS has antibacterial properties but has been linked to thyroid dysfunction and increased antibiotic resistance. Parabens and musks bioaccumulate in fish tissues, leading to oxidative stress and alterations in reproductive behavior. Due to their low volatility and persistence, PCPs accumulate in sewage sludge and then enter agricultural soils when biosolids are used as fertilizers, constituting a secondary source of pollution [62]. The environmental risk posed by PCPs stems from their chronic effects on the environment, even at low concentrations, and from their potential synergistic interactions with pharmaceuticals and other CECs. As a result, PCPs have attracted significant research attention, particularly regarding their degradation by AOPs such as activated PAA. Table 7 summarizes the results of studies that have removed PCPs using various PAA activators.

The compounds tested included triclosan (TCS), benzophenone (BPh), 3-(4-methylbenzylidene)camphor (4MBC), oxybenzone (OXB), and N,N-diethyl-m-toluamide (DEET). Zhou et al. [84] investigated the removal of TCS, a commonly used antimicrobial agent known to be stable in aqueous environments, using CoFe_2_O_4_ as the activator. In this case, 0.8 mM PAA combined with 0.5 g/L CoFe_2_O_4_ at pH 7.0 achieved 90.3% degradation in just 10 min. In contrast, Piekutin et al. [98] present UV/PAA treatment for DEET removal (43.7–182.8 µg/L) using 0.1425 mL of a 16% PAA solution, achieving variable efficiencies of 70–100% after 5–10 min, demonstrating a strong dependence on exposure conditions. Kotowska et al. show that for UV-filtering compounds such as BPh, 4MBC, and OXB (100 µg/L each), homogeneous activation of Fe^3+^ was highly effective [88]. Under conditions of 10.5 mg/L PAA, 10^−3^ mol/L Fe*^3+^*, and acidic pH (3.0), these compounds were almost completely removed (95%) within 90 min. Both homogeneous (activated Fe*^3+^*) and heterogeneous (CoFe_2_O_4_) catalysts provided high efficiency, though the latter enabled significantly shorter reaction times. Compared with pharmaceuticals, PCP degradation via PAA activation appears to depend more on the catalyst type than on the oxidant dose itself. The highest efficiency and shortest reaction times were observed for heterogeneous systems such as CoFe_2_O_4_/PAA, where triclosan removal reached over 90% within 10 min at neutral pH. This suggests that catalytic selectivity and efficient generation of reactive organic radicals play a decisive role for compounds with stable aromatic and phenolic structures. Homogeneous Fe^3+^/PAA systems were also highly effective for UV filters such as benzophenone, oxybenzone, and 4MBC, achieving degradation of approximately 95%, although acidic conditions (pH 3.0) and longer reaction times were required. In contrast, UV/PAA treatment showed greater variability in efficiency and was strongly dependent on irradiation intensity and compound photoreactivity, as observed with DEET. From an implementation perspective, heterogeneous catalysts offer better operational feasibility due to catalyst recovery and near-neutral pH conditions, whereas homogeneous systems may be limited by iron dosing and post-treatment requirements. A major unresolved issue remains the identification of transformation products, especially in the case of endocrine-disrupting PCPs, where partial oxidation may not eliminate biological activity. Therefore, future studies should focus not only on removal efficiency but also on residual toxicity and long-term environmental safety.

#### 4.2.3. Pesticides

Pesticides include herbicides, insecticides, and fungicides that are intentionally released into the environment for agricultural or public health purposes. Classes such as organophosphates (chlorpyrifos (CPF), diazinon (DZN)), carbamates (carbaryl (CBL), carbendazim (CBD)), triazines (atrazine (ATZ), simazine (SMZ)), and pyrethroids (cypermethrin (CYP), bifenthrin (BFT)) are among the most frequently detected in aquatic systems [5,99]. Their occurrence results from surface runoff, leaching, atmospheric deposition, and wastewater treatment plant effluents [72]. Many modern pesticides are designed for chemical stability and specificity, but these same characteristics lead to environmental persistence and bioaccumulation. Numerous studies confirm their ability to disrupt endocrine systems by interacting with glucocorticoid and estrogen receptors, altering metabolic and reproductive processes in both aquatic and terrestrial fauna [62,97]. For example, AZT is associated with feminization in amphibians, while organophosphates inhibit acetylcholinesterase activity in fish and invertebrates. Pesticide residues in sediments can constitute a long-term source of pollution, gradually being released into the water column and affecting food webs. Despite existing regulations, emerging pesticide metabolites and their transformation products are often not monitored, yet they exhibit toxicity comparable to or even higher than that of the parent pesticides. Literature data demonstrate the use of activated PAA in removing this type of contaminant (Table 8).

The compounds tested included atrazine (AZT), mecoprop (MCPP), diazinon (DZN), and two endosulfan isomers (ESI and ESII). The study by Al Umairi et al. [79] tested AZT, MCPP, and DZN at 50 µg/L, all exposed to UV/PAA systems with 3.2 mg/L PAA and 2.3 mW/cm^2^ UV irradiation at near-neutral pH (7.2). Although the data indicate treatment effectiveness, the precise removal efficiency was not reported in the source. Zhou et al. [83] also tested AZT under heterogeneous catalysis, where CoFe_2_O_4_/PAA (5 µM pesticide, 0.8 mM PAA, 0.5 g/L catalyst, pH 7.0) degraded only 9.2% after 10 min. CoFe_2_O_4_/PAA system showed selectivity toward the degradation of organic contaminants. In contrast, in a paper by Kotowska et al. [88], the PAA/Fe^3+^ system was highly effective against more persistent pesticides such as ESI and ESII (100 µg/L each). Under acidic conditions (pH 3.0), at a PAA concentration of 10.5 mg/L and a Fe^3+^ concentration of 10^−3^ mol/L, both isomers achieved 95% elimination within 90 min. In the case of pesticides, the efficiency of PAA-based oxidation depends primarily on molecular structure rather than solely on oxidant concentration. Hydrophobic and persistent compounds, such as endosulfan isomers, responded well to Fe^3+^/PAA treatment, achieving approximately 95% elimination under acidic conditions, confirming the suitability of this system for more recalcitrant organochlorine pesticides. On the other hand, the heterogeneous CoFe_2_O_4_/PAA showed very limited efficacy against atrazine (only 9.2%), indicating that steric hindrance and halogen substitution can significantly reduce susceptibility to oxidation by organic radicals. UV/PAA systems appear more suitable for moderately persistent herbicides and insecticides, such as mecoprop or diazinon, although reported studies often lack precise data on mineralization. These findings indicate that there is no single activation strategy that can be universally applied to all classes of pesticides. Process optimization must therefore consider both the chemical composition of the pesticides and the composition of the aqueous matrix. An additional limitation is the insufficient assessment of transformation products, as partial oxidation can yield metabolites with toxicity comparable to, or even higher than, that of the parent pesticide. In practice, this issue is as important as removal efficiency itself.

#### 4.2.4. Industrial Chemicals and By-Products (ICBs)

This category encompasses synthetic compounds and unintended byproducts generated during industrial production, combustion, and waste management. Key examples include perfluoroalkyl substances (PFAS) and polyfluoroalkyl substances (PFAS), plasticizers (phthalates), bisphenol A (BPA), flame retardants (PBDEs, organophosphates), dioxins, furans, heavy metals, and nanomaterials [62,72,73]. Due to their hydrophobicity and chemical inertness, these substances exhibit strong sorption to organic matrices and long-range atmospheric and hydrological transport. PFAS are emblematic “forever chemicals” characterized by exceptional persistence due to the strength of their C-F bonds. They bioaccumulate at all trophic levels, affect lipid metabolism, and have been linked to immunotoxic and carcinogenic effects in humans [73]. Similarly, brominated flame retardants and PCBs disrupt thyroid function and neurodevelopment, while phthalates and BPA act as estrogenic agents. Industrial wastewater, landfill leachate, and e-waste recycling are the main sources of ICBs. Their global distribution, including detection in polar snowpacks and deep-sea sediments, highlights their mobility and resistance to natural degradation [62,72,73]. Improper disposal can contribute to the accumulation of toxic compounds in the environment, potentially disrupting natural cycles and affecting the food chain. Therefore, the search for new effective procedures for treating ICBs wastewater is crucial [73]. Table 9 shows research studies demonstrating the use of PAA as a promising oxidant for ICBs.

**Figure 2:**
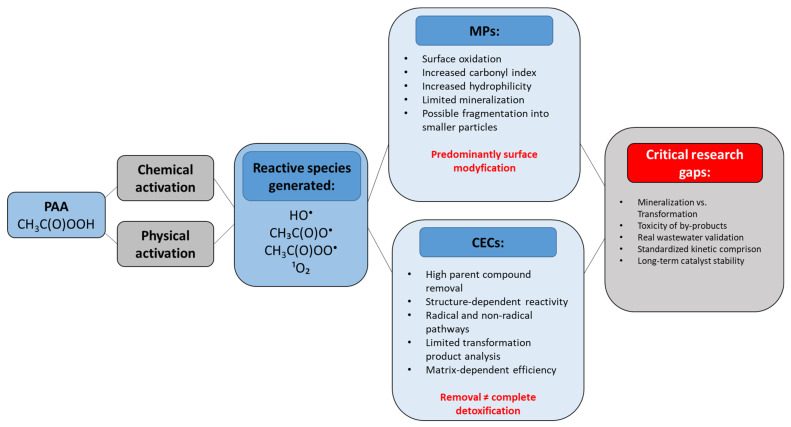
Conceptual framework illustrating activation pathways of PAA, generation of reactive oxygen species, and their differential effects on MPs and other CECs.

Among the various pollutant categories studied, industrial chemicals appear particularly well suited for removal by PAA activation, with numerous examples demonstrating near-complete degradation under optimized conditions. Wu et al. [100] presented a study in which the azo dye Orange G was completely degraded (100%) within 90 min when Co_3_O_4_ was used as an activator (0.1 g/L catalyst, 0.5 mM PAA). In the case of BPA, various types of PAA activators have been reported in the literature for its removal. The Co^2+^/PAA system presented in Kim et al. [56] achieved 87.7% removal within 30 minutes at moderate concentrations (100 µM PAA, 10 µM Co^2+^, pH 7.1). Under similar conditions, only at pH 3.5 was satisfactory BPA removal achieved in the work of Liu et al. [85], and at the same time, PMSO (100 µM PAA, 10 µM Co^2+^, pH 3.5) showed significant removal within 30 min. Furthermore, iodide-activated PAA (I^−^/PAA, 500 µM PAA with 100 µM I^−^) presented in the work of Zou et al. [101] completely removed BPA in just 10 minutes, demonstrating the strong synergistic potential of halide activators. The research presented by Kiejza et al. [105] concerns the use of nickel cobaltite nanoparticles (NiCo_2_O_4_ NPs) for the heterogeneous activation of PAA and application of the NiCo2O4-PAA system for the degradation of 10 organic micropollutants. The optimal amount of catalyst (115 mg/L), PAA concentration (7 mM), and pH (7) catalyzed the almost complete degradation (99.9%) of a bisphenol mixture within 10 minutes. In a subsequent study, Kiejza et al. [104] examined in detail the degradation of 10 bisphenols (BPs). These included bisphenol F (BPF), bisphenol E (BPE), bisphenol A (BPA), bisphenol C (BPC), bisphenol B (BPB), bisphenol G (BPG), bisphenol Cl_2_, bisphenol Z, bisphenol AP (BPAP), and bisphenol M (BPM). The study analyzed Fe^3+^-SAc/PAA systems (where SAc is salicylic acid acting as a chelating agent). In the Fe^3+^/SAc/PAA system, the optimal conditions included a PAA concentration of 5 mM, Fe^3+^ of 0.5 mM, SAc of 1 mM, and an initial pH of 6. This system proved significantly more efficient, achieving almost 100% degradation of all 10 bisphenols within 10 minutes. Additional metal-based systems, presented by Rabbil et al. [107], such as MK-AAFs-Co^2+^/PAA also performed well, consistently achieving >90% contaminant removal in a short time (10–15 min). In this study, alkali-activated aluminosilicate foams (AAFs) were compared as supports for Co^2+^ and tested for aqueous phenol abatement. Other industrial contaminants, such as rhodamine B (RhB), bisphenol AF (BPAF), 2-chlorophenol (2-CP) and aniline, present in study by Zhou et al. [84] were also successfully removed using CoFe_2_O_4_/PAA, conditions 0.8 mM PAA, 0.5 g/L CoFe_2_O, pH 7.0 although efficiencies varied (from 50% to >95%) in 10 min, reflecting differences in the contaminant structure and reactivity. Carbon-based materials such as carbon nanotubes (CNTs) have also been extensively tested. In the study of Miao et al. [102] their combination with PAA (0.25 mM PAA, 0.2 g/L CNTs) provided a wide range of applications. High efficiencies (57–100%) were reported for various phenolic contaminants, including 4-chlorophenols (4-CP), 4-nitrophenols (4-NP), 2-methoxyphenols (2-MOP), 2,4-dichlorophenols (2,4-DCP), phenol (PE), and also BPA, confirming the versatility and effectiveness of CNTs as activators. Compared with pharmaceuticals and other CECs, only a limited number of studies have investigated PFAS degradation using activated PAA systems. Existing studies primarily focus on PFOA. The available literature indicates that catalytic activation using Co- and Fe-based materials significantly enhances PFAS degradation, whereas non-activated PAA exhibits negligible reactivity toward these highly persistent compounds. Heterogeneous catalysts, particularly Co–Fe-supported biochar systems, demonstrated the highest degradation and defluorination efficiencies. However, complete mineralization is rarely achieved, and the formation of shorter-chain fluorinated intermediates remains a major challenge. Among the CEC groups analyzed, industrial chemicals and byproducts appear to be the most susceptible to PAA-based oxidation, particularly in systems activated with transition metals and carbon materials. Compounds such as bisphenol A, phenols, orange G, and rhodamine B were often removed with efficiency close to 100%, especially in Co-based systems, including Co_3_O_4_/PAA, Co^2+^/PAA, and NiCo_2_O_4_/PAA. This high susceptibility is mainly due to the presence of electron-rich aromatic structures that readily react with the CH_3_COOO• and CH_3_COO• radicals generated during PAA activation. Carbon-based catalysts such as CNT/PAA have also shown promising performance, offering an alternative pathway based on electron transfer and enhanced stability in more complex matrices. Despite these favorable results, the treatment of highly persistent contaminants such as PFAS remains significantly more challenging because the exceptional stability of the C-F bonds severely limits oxidation efficiency even in advanced systems. Furthermore, catalyst durability, cobalt leaching, and the risk of secondary contamination remain significant obstacles to scale-up. Another important aspect is that high degradation rates often refer only to the disappearance of the parent compound, while the toxicity and persistence of oxidation byproducts are still poorly understood. Therefore, future research should go beyond laboratory removal rates and include a full environmental risk assessment under realistic wastewater conditions. The Figure 2 highlights the predominance of surface modification in MPs and parent compound removal in CECs studies, as well as key research gaps identified in the literature.

### 4.3. Implications for Process Design and Interpretation of PAA Performance

The comparative analysis of the studies summarized in Table 6, Table 7, Table 8 and Table 9 show that activated PAA is a versatile and promising method for eliminating CECs from water and wastewater. The wide range of possible activation pathways enables process optimization based on pollutant type and environmental conditions, making PAA a promising technology for sustainable water treatment. The observed variability across compound classes and activation methods reflects not only differences in catalytic activity but also fundamental disparities in oxidation pathways and pollutant susceptibility. Importantly, the data reveal that similar removal efficiencies may arise from different mechanistic regimes, which has direct implications for process stability and scalability. Systems that degrade rapidly under controlled laboratory conditions may exhibit reduced robustness in realistic water matrices, whereas slightly slower systems may exhibit greater resilience to background constituents. Another key observation is that pollutant responses to PAA activation appear to cluster by structural features rather than regulatory classification (e.g., pharmaceutical vs. pesticide). Electron-rich aromatic compounds tend to be consistently susceptible across activation strategies, while sterically hindered or halogenated molecules display more selective degradation patterns. These findings suggest that the selection of activation strategy should be guided by the anticipated composition of the contaminant mixture rather than by performance toward individual model compounds. From an engineering perspective, this underscores the importance of aligning activation pathways with treatment objectives, whether prioritizing rapid transformation, matrix tolerance, or operational simplicity.

From a practical implementation perspective, PAA-based wastewater treatment should also be assessed in terms of effluent quality and operating costs. Because PAA decomposes mainly into acetic acid, hydrogen peroxide, oxygen, and water, residual acetic acid may add a biodegradable organic load and slightly increase the biochemical oxygen demand (BOD) of the final effluent, particularly at higher PAA doses or when post-treatment residence time is insufficient. However, this effect is usually limited under typical wastewater treatment conditions because acetic acid is readily biodegradable. In addition, PAA is generally more expensive than sodium hypochlorite at the reagent level, which may limit its use in large-scale applications. Nevertheless, this disadvantage should be considered alongside other process-related factors, such as the absence of chlorine residuals, the need for no separate dechlorination step, a lower risk of chlorinated disinfection by-product formation, and differences in storage, handling, and dosing requirements. Therefore, the feasibility of PAA should be evaluated on a site-specific basis, taking into account both reagent costs and overall operational performance.

## 5. Effect of Matrix on the Oxidation of CECs in the PAA System

The influence of water matrix components on PAA-based oxidation extends beyond simple radical scavenging effects. Recent studies indicate that inorganic ions and natural organic matter can alter not only the reaction rate but also the dominant oxidation pathway. As a result, the same activation system may operate through different mechanisms depending on matrix composition. This shift in mechanistic regime is particularly relevant for translating laboratory findings into realistic water and wastewater treatment scenarios. The chemical and physical properties of the solution, as well as the presence of inorganic anions, cations, and natural organic matter (NOM), significantly influence the PAA activation mechanisms, the generation of reactive oxygen species (ROS), and their contribution to the degradation of organic micropollutants [46,91,103]. Studies conducted across various PAA activation systems have observed significant differences in oxidation efficiency depending on matrix composition, clearly indicating the need to consider environmental factors when designing and interpreting AOPs [73,105]. Matrix components that influence the process include bicarbonate and carbonate ions (HCO_3_^−^/CO_3_^2−^), which act as scavengers for HO• radicals, converting them to less reactive carbonate radicals (CO_3_^•−^). This typically leads to reduced oxidation efficiency, though under alkaline conditions (pH ≈ 8.5), CO_3_^•−^ radicals can participate in the degradation of electron-rich compounds such as DCF. Importantly, carbonate species do not merely inhibit oxidation but may redirect it toward more selective pathways involving carbonate radicals. While less reactive than hydroxyl radicals, these species can sustain oxidation of electron-rich contaminants, potentially improving transformation selectivity under alkaline conditions. Chloride ions (Cl^−^) react with HO• and CH_3_COOO• radicals to form less reactive chlorine radicals (Cl•, Cl_2_^•−^, ClOH^•−^) [42]. This effect is particularly noticeable in catalytic systems such as Fe^2+^-PAA or NiCo_2_O_4_-PAA, where BPs removal efficiency can decrease by up to 70%. In some systems, chloride ions may also facilitate alternative oxidation pathways by forming reactive halogen species. This indicates that chloride presence should not be interpreted solely as inhibitory, but rather as a factor that can modify oxidation selectivity.

In contrast, sulfate ions (SO_4_^2−^) usually do not affect the process, while nitrate ions (NO_3_^−^) may moderately support oxidation by generating HO• radicals under the influence of UV radiation [104,105,110]. Natural organic matter, including humic acids, exhibits strong inhibitory effects, reacting with radicals and forming stable complexes with metal ions (Fe^2+^, Co^2+^), which limits their ability to activate PAA [100,104,106]. In photoactivated systems (UV/PAA), NOM can additionally absorb UV radiation, reducing the efficiency of PAA photolysis [46,110]. Beyond acting as a radical sink, NOM may compete with target pollutants for catalytic sites or participate in electron-transfer processes. This dual role highlights its capacity to either suppress or stabilize oxidation pathways depending on system configuration. Similar effects are observed in the presence of transition-metal ions; for example, Cu^2+^ can form inactive complexes or precipitate insoluble species, although under certain conditions it can also catalyze PAA decomposition and generate additional radicals. In summary, the composition of the aqueous matrix significantly influences the mechanisms and efficiency of PAA-based oxidation processes. Bicarbonates, chlorides, and NOM most often act as inhibitors, but some ions can, under certain conditions, support the process by generating additional reactive oxygen species. Although many PAA-based systems demonstrate contaminant removal efficiencies exceeding 90–100% in the laboratory, directly translating these data to full-scale water treatment remains difficult. Most studies are conducted using ultrapure water, single-component solutions, and controlled pH conditions, which do not reflect the real-world complexity of wastewater. In practical systems, natural organic matter (NOM), bicarbonate, chloride, and suspended solids can significantly reduce oxidation efficiency by scavenging reactive radicals or deactivating catalytic sites. Furthermore, catalyst stability, metal leaching, regeneration efficiency, fouling, and operating costs are often underestimated. High parent compound degradation efficiencies also do not necessarily indicate complete mineralization or detoxification, as transformation products may remain persistent or even more toxic. Therefore, future studies should prioritize validation in real-world matrices, catalyst recyclability, toxicity assessment, and techno-economic evaluation, rather than relying solely on laboratory-scale removal percentages. To summarize these effects in different PAA systems, Table 10 shows the influence of the main components on the degradation efficiency and mechanistic effects.

## 6. Study of the Degradation Mechanism of CECs and MPs

Degradation of organic compounds by AOPs occurs primarily via reactive radical species, the type and role of which depend on the peroxyacid used and its activation method. In the case of PAA, the unstable peroxide bond (-O-O-) with a low dissociation energy (~170 kJ·mol^−1^) is crucial and easily homolytically cleaved, leading to the formation of HO• and CH_3_COO• radicals [24,42]. Due to the low rate of spontaneous PAA decomposition, this process requires the use of activators. UV radiation initiates the decomposition of PAA, generating CH_3_COO• and HO^∙^ radicals, while simultaneously activating the H_2_O_2_ present in the system, which is in equilibrium with PAA. In light-activated systems, such as UV/PAA or PAA/sunlight, both organic radicals and HO• play crucial roles, co-responsible for the decomposition of organic compounds [46,103,111]. Activation of PAA by transition-metal ions proceeds via redox cycles that generate reactive radicals. In the Fe^2+^/PAA system, the main source of HO• is PAA decomposition, and this process is more efficient than the classical Fenton system (Fe^2+^/H_2_O_2_). The reaction produces CH_3_COO•, HO• radicals, and ferryl ions (Fe^4+^O^2+^) [87,92,104]. In the Co^2+^/PAA system, CH_3_COOO• and CH_3_COO• radicals predominate, whereas in the Fe^3+^/PAA system, selective CH_3_COO•, CH_3_C(O)OO•, CH_3_CO• radicals are generated, of which organic radicals are the most important [87,104]. The addition of a chelating agent, such as salicylic acid (SAc), stabilizes Fe^3+^ ions in solutions with pH up to 6, and in the Fe^3+^–SAc–PAA system, the main oxidizing agents are high-valent iron species (Fe^4+^), with a minor contribution of organic radicals [104]. In heterogeneous systems, such as NiCo_2_O_4_/PAA, the degradation of organic compounds occurs mainly via superoxide radicals (O_2_^•−^), while the contribution of HO• is marginal [105]. Table 11 shows the characteristics of the main radicals and reactive species formed in PAA-based AOPs.

Oxidation reactions produce lower-molecular-weight products that are less toxic and more biodegradable. Studies of BPs oxidation in UV/PAA systems indicate gradual degradation of the molecules, occurring primarily through hydroxylation and oxygenation of aromatic rings [103]. In some cases, decarboxylation, as in the case of IBU, and cleavage of aliphatic chains leading to the formation of lower-molecular-weight compounds have also been observed. Identified BPs oxidation products include derivatives with *m*/*z* 273, 306, 313, 320, 352, and 354, resulting from the addition of hydroxyl groups. In the case of dichlorobisphenol (BPCl_2_), dechlorination products with *m*/*z* 212 and 246 have been detected. Higher-molecular-weight compounds, such as BPM, can also produce higher-molecular-weight products. Similar mechanisms have been observed for other organic micropollutants. CBZ, IBU, and NPX undergo hydroxylation and oxygenation in UV/PAA systems, generating numerous derivatives (17, 11, and 30 products, respectively) [81]. In the case of SMX activated with the Co/PAA system, products of hydroxylation, side-chain oxidation, and coupling are formed, such as nitroso-, hydroxy-, nitro-, and azo-SMX [106]. DCF in the Fe^2+^/PAA system produces products with *m*/*z* 312 and 328 resulting from hydroxylation, *m*/*z* 147 from C–N bond cleavage, and *m*/*z* 122 resulting from decarboxylation and dehydrogenation. The reaction mechanisms and identification of reactive species were investigated using a combination of experimental, analytical, and computational techniques. Selective scavengers were used to confirm the participation of specific radicals: *t*-butanol (TBA), which reacts exclusively with HO•; methanol (MeOH), which interacts with both ∙OH and organic radicals; *p*-benzoquinone, which identifies the presence of O_2_^•−^; sodium azide, which detects ^1^O_2_; and benzoic acid (BA), which was used to confirm Fe^IV^ activity [42]. The addition of TBA in the UV/PAA system decreased the degradation efficiency, indicating the key role of HO•. Whereas in the NiCo_2_O_4_/PAA system, its effect was negligible, suggesting the dominance of superoxide radicals. Electron paramagnetic resonance (EPR) spectroscopy was used to confirm the radical nature of the oxidation processes and identify spin adducts using FDMPO and DMPO radical traps. FDMPO/HO• and FDMPO/•CH_2_OH adducts were detected in the studied systems, confirming the generation of hydroxyl and carbon-centered radicals. The highest EPR signal intensities were observed in the PAA/Fe^2+^ and PAA/Fe^3+^ systems, whereas in the Fe^3+^–SAc–PAA system, the number of HO• was low, indicating a significant role of Fe(IV) as the main oxidant [104]. Identification of reaction products was performed using advanced chromatographic and spectrometric techniques, including gas chromatography-mass spectrometry (GC-MS), liquid chromatography-mass spectrometry (LC-MS), and electrospray ionization-mass spectrometry (ESI-MS). Analyses of the post-reaction mixtures revealed products consistent with hydrogen abstraction (H-abstraction), HO• addition, and aromatic ring degradation [62,72]. To gain insight into the reaction at the molecular level, density functional theory (DFT) calculations using the M06-2X functional were performed, which showed that the main pathways initiating the degradation are hydrogen abstraction and ∙OH addition, followed by β-cleavage. Energy barrier analysis confirmed that the energy provided by visible light (~60 kcal/mol) is sufficient to initiate the reaction, but effective generation of hydroxyl radicals requires UV radiation [111].

MPs degradation in PAA-based systems is primarily regulated by radical-mediated oxidative processes. Upon activation (e.g., in UV- or H_2_O_2_-assisted systems), PAA generates reactive species, which initiate hydrogen abstraction and electrophilic attack on polymer chains [69,70]. These reactions promote the formation of oxygen-containing functional groups (e.g., carbonyl and hydroxyl groups), thereby increasing surface oxidation and polarity. In more susceptible polymers, limited chain scission may occur, accompanied by changes in crystallinity and surface morphology. However, available evidence suggests that oxidative modification is largely confined to the polymer surface, with insufficient evidence of extensive backbone cleavage or complete mineralization [68,69].

## 7. Conclusions

PAA has emerged as a versatile oxidant in AOPs, capable of generating a broad spectrum of reactive radicals, including HO•, CH_3_C(O)O•, CH_3_CO•, and CH_3_COOO•. These compounds drive the degradation of persistent CECs beyond the capabilities of conventional treatment. The choice of activator is crucial, and transition metals (Co, Fe, Mn), metal oxides (Co_3_O_4_, NiCo_2_O_4_, CoFe_2_O_4_), composites, carbon materials, UV radiation, and halide activation have all been shown to enhance radical production and reduce micropollutants. Current data indicate that cobalt- and iron-based systems, as well as carbon and photo-assisted activation, are particularly effective pathways. Despite the promising performance of PAA-based systems, several critical challenges remain unresolved. Most available studies focus primarily on parent compound removal, while limited attention is given to complete mineralization, transformation product identification, and toxicity assessment. In the case of MPs, oxidative surface modification appears to prevail over full polymer degradation, raising questions about the actual environmental benefit of the process. Furthermore, the lack of standardized kinetic evaluation and the limited number of studies conducted in real wastewater matrices hinders direct comparison of reported efficiencies and practical implementation. Future research should therefore prioritize mechanistic clarification of radical and non-radical pathways, systematic assessment of mineralization and by-product toxicity, and validation under environmentally relevant conditions. Addressing these aspects will be essential to determine the true sustainability and applicability of PAA-based advanced oxidation processes in water and wastewater treatment. Full-scale assessments should also include the possible contribution of residual acetic acid to effluent BOD and the higher reagent cost of PAA compared with sodium hypochlorite, balanced against reduced dechlorination requirements, lower formation of chlorinated by-products, and simplified operational management.

## Figures and Tables

**Table 1:** Chemical and physical properties of PAA [19].

Name	Peracetic Acid (PAA)
Chemical formula	C_2_H_4_O_3_
Molar Mass (g/mol)	76.05
Density (g/mL)	1.04
Boiling point (°C)	110
Melting point (°C)	0.2
Acidity (pKa)	8.2

**Table 2:** Summary of oxidation potentials of common oxidants used in water treatment processes [19,24,41].

Oxidant	Oxidation Potential (V)
Hydroxyl radical	2.8
Sulfate radical	2.5–3.1
Ozone	2.1
Persulfate	2.0
PAA	1.0–1.9
Peroxymonosulfate	1.8
H_2_O_2_	1.8
Potassium permanganate	1.7
Chlorine dioxide	1.5
Chlorine	1.4

**Table 3:** Practical trade-offs between physical and chemical PAA activation strategies.

Strategy	Main Strengths	Main Constraints	Best Suited for
Physical activation: UV, US, thermal	No catalyst addition; lower risk of secondary contamination; operationally simple concept	Continuous energy input; low selectivity; reduced efficiency in complex matrices due to radical scavenging, turbidity, NOM, and inorganic ions	Relatively clean or transparent waters; tertiary effluents; systems where catalyst addition is undesirable
Homogeneous metal activation	Fast reaction kinetics; efficient catalytic redox cycling; strong activation under controlled conditions	pH dependence; metal residuals; difficult catalyst recovery; possible secondary contamination	Controlled treatment systems where pH and residual metals can be managed
Heterogeneous metal activation	High catalytic efficiency; easier separation than homogeneous systems; potential reusability	Metal leaching; catalyst deactivation; recovery and stability requirements; synthesis cost	Systems requiring high efficiency and catalyst reuse, provided leaching is controlled
Carbon-based activation	Metal-free or low-metal option; better matrix tolerance; tunable surface chemistry; possible non-radical pathways	Often slower kinetics; performance depends on surface properties; catalyst recovery still required	Complex matrices where radical scavenging limits conventional radical-based oxidation

**Table 4:** The observed changes in polyethylene (PE), polypropylene (PP), and polystyrene (PS) properties after UV/PAA treatment. Author’s own elaboration based on [67].

Property	MPs	Initial	After UV/PAA	Effect
Morphology & Surface	PE	Rough surface, cracks, pits	Rough surface, cracks, pits	Surface became rough with cracks and pits; particle size decreased
	PP	Rough surface, cracks, pits	Rough surface, cracks, pits	Particle size slightly decreased
	PS	White color	Light yellow	Color change due to the UV sensitivity of aromatic rings
Average Particle Size (μm)	PE	104.3	35.0	Significant size reduction
	PP	123.9	111.7	Moderate size reduction
	PS	121.9	119.8	Slight size reduction
BET Surface Area (m^2^ g^−1^)/Micropore Volume (cm^3^ g^−1^)	PE	3.73/0.003	2.07/0.002	Decrease in both, contrary to the general AOP trend
	PP	1.43/0.001	2.88/0.003	Increase observed
	PS	1.16/0.001	53.77/0.052	Significant increase
Hydrophobicity (Water Contact Angle, °)	PE	122.2	131.4	Increased hydrophobicity, contrary to the general AOP effect
	PP	122.4	113.4	Decreased hydrophobicity
	PS	124.8	110.6	Decreased hydrophobicity
Crystallinity (%)	PE	23.7	41.7	Increased crystallinity
	PP	51.5	61.0	
	PS	45.9	36.6	Decreased crystallinity
2-Nitrofluorene adsorption (μg g^−1^)	PE	597.9	607.2	Adsorption increased; equilibrium reached in 24 h
	PP	599.6	604.9	
	PS	599.8	609.7	

**Table 5:** Main effect of PAA/H_2_O_2_ on microplastics. The author’s own elaboration is based on [68].

Microplastic	Main Radicals (from PAA/H_2_O_2_ System)	Oxidation Products/Chemical Changes	Surface and Mechanical Effects
PE	HO•, CH_3_COO•	no specific products identified; partial surface oxidation	initial swelling (~0.09%), then slow dissolution; mass loss and decreased mechanical properties (K_0_ ≈ 0.03%/h, K_6_ ≈ 0.025%/h after 1000 h)
PVC	HO•, CH_3_COO• (stronger PAA activation than PE)	no specific products identified; surface degradation	initial swelling (~0.11%), followed by dissolution; mass loss; stronger activation of PAA dissociation than PE; K_0_ ≈ 0.04%/h, K_6_ ≈ 0.05%/h after 1000 h
PTFE	practically non-reactive toward PAA/H_2_O_2_	no chemical changes	high resistance; no swelling, dissolution, or loss of mechanical properties (stable up to 1000 h)

**Table 6:** Summary of studies on the use of various activators for PAA activation in pharmaceuticals removal.

Pharmaceuticals	PPCPs Conc.	Processes	PAA Conc.	Catalyst Dosage/ Activator	pH	Reaction Time	Removal Efficiency (%)	Ref.
Amoxicilin (AMX)	25 mg/L	UV-C/PAA	0.1 mM	254 nm	not reported	2 h	78	[74]
Sulfamethoxazole (SMX)	50 µM	LaCoO_3_/PAA	660 µM	20 mg/L	7.0	1 h	Effective degradation	[75]
	10 µM	CoFe_2_O_4_/PAA	200 µM	0.1 g/L	7.0	30 min	87.3	[76]
	5 µM	Co-CN/PAA	0.1 mM	50 mg/L	6.5	350 s	98.5	[77]
	15 µM	Co^2+^/PAA	100 µM	10 µM	7.1	30 min	87.2	[56]
	50 µg/L	UV/PAA	3.2 mg/L	UV 2.3 mW/cm^2^	7.2	not reported	not reported	[78]
	10 µM	PAA/Br^−^	0.2 mM	0.2 mM	7.1	1 h	81.3	[79]
	1 mg/L	MOF-(Fe1,Co1)/PAA	10 mg/L	25mg/L	not reported	60 min	99.7	[80]
	5 mg/L	UVA/PAA	50 mg/L	360 nm	not reported	30 min	34	[81]
	5 µM	Fe^2+^-zeolite/PAA	400 µM	0.8 g/L	7.0	50 min	100	[82]
	5 µM	CoFe_2_O_4_/PAA	0.8 mM	0.5 g/L	7.0	10 min	97.1	[83]
	5 µM	ZVCo/PAA	50 µM	0.1 g/L	7.0	5 min	99	[84]
	10 µM	Co^2+^/PAA	100 µM	10 µM	3.5	30 min	not reported	[85]
	0.079 mM	AC600/PAA	0.26 mM	50 mg/L	7.0	150 min	99.4	[86]
	10 µM	Fe^3+^/MoS_2_/PAA	0.3 mM	0.1 mM Fe^3+^ 0.1 g/L MoS_2_	3.0	15 min	97.8	[87]
17α-ethinylestradiol (EE2)	10 µM	PAA/Br^−^	0.2 mM	0.2 mM	7.1	1 h	48.7	[79]
Estrone (E1)	100 µg/L	PAA/Fe^3+^	10.5 mg/L	1 mM	6.0	90 min	90	[88]
Diethylstilbestrol (DSB)	100 µg/L	PAA/Fe^3+^	10.5 mg/L	1 mM	6.0	90 min	90	
Naproxen (NPX)	10 µM	PAA/Br^−^	0.2 mM	0.2 mM	7.1	1h	31.3	[79]
	15 µM	Co^2+^/PAA	100 µM	10 µM	7.1	30 min	93.4	[56]
Carbamazepine (CBZ)	15 µM	Co^2+^/PAA	100 µM	10 µM	7.1	30 min	61.8	[56]
	1 mg/L	PAA/BC-CoFe_2_O_4_	0.8 mM	0.3 g/L	7.0	not reported	100	[89]
	1 mg/L	PAA/BC-MnFe_2_O_4_	0.8 mM	0.3 g/L	7.0	not reported	7	[89]
	1 mg/L	PAA/BC-CuFe_2_O_4_	0.8 mM	0.3 g/L	7.0	not reported	7	[89]
	50 µg/L	PAA	9.6 mg/L	not reported	7.2	not reported	<25	[78]
	1 mg/L	MOF-(Fe1,Co1)/PAA	10 mg/L	25 mg/L	not reported	60 min	97.1	[80]
	10 µM	Co^2+^/PAA	100 µM	10 µM	3.5	30 min	not reported	[85]
Fluconazole (FCL)	50 µg/L	PAA	9.6 mg/L	not reported	7.2	not reported	<25	[78]
Trimethoprim (TMP)	50 µg/L	PAA	9.6 mg/L	not reported	7.2	not reported	25–50	[78]
Sulfadiazine (SDZ)	10 mg/L	UV/PAA/NO_2_^−^	10 mg/L	2.12 mW/cm^2^	7.0	10 min	not reported	[90]
Acetaminophen (ACT)	20 mg/L	PAA/UVc-LED/Fe^2+^	4 mM	0.5 mM	5.0	30 min	95	[91]
Bezafibrate (BZF)	1 mg/L	MOF-(Fe1,Co1)/PAA	10 mg/L	25mg/L	not reported	60 min	98.1	[80]
Tetracycline (TC)	10 µM	nZVI/PAA	100 µM	0.06 g/L	6.0	30 min	>95	[92]
Ibuprofen (IBU)	1 mg/L	MOF-(Fe1,Co1)/PAA	10 mg/L	25mg/L	not reported	60 min	74.9	[80]
Moxifloxacin (MOX)							99.5	
Propranolol (PPL)							99.8	
Diclofenac (DCF)							97.1	
Diclofenac (DCF)	5 µM	(CoFe_2_O_4_)/PAA	0.8 mM	0.5 g/L	7.0	10 min	100	[83]
	5 µM	PBS/PAA	0.55 mM	0.1 M	7.4	50 min	95.7	[93]
	1 µM	ZVC/PAA	100 µM	0.5 g/L	2.0	40 min	100	[94]
Chlortetracycline (CTC)	10 µM	Fe^2+^/PAA	100 µM	0.06 g/L	6.0	5 min	>95	[92]
Oxytetracycline (OTC)						5 min		
Cefalexin (CFX)						30 min		
Ampicillin (AMP)						30 min		
Sulfamethazine (SMZ)	5 mg/L	ABC/PAA	5 mg/L	0.1 g/L	7.0	100 min	72.8	[95]

Abbreviations: MOF-(Fe1,Co1)—bimetallic MOF containing Fe and Co in a 1:1 molar ratio; ZVCo—Zero-Valent Cobalt; AC600—Activated Carbon prepared at 600 °C; BC-AFe_2_O_4_—biochar-supported spinel ferrites AFe_2_O_4_ (A = Cu, Co, or Mn); PBS—Phosphate-Buffered Saline; ZVC—Zero-Valent Copper; ABC—Activated Biochar.

**Table 7:** Summary of studies on the use of various activators for PAA activation in personal care products removal.

Personal Care Products	PCPs Conc.	Processes	PAA Conc.	Catalyst Dosage/ Activator	pH	Reaction Time	Removal Efficiency (%)	Ref.
Triclosan (TCS)	5 µM	(CoFe_2_O_4_)/PAA	0.8 mM	0.5 g/L	7.0	10 min	90.3	[83]
Benzophenone (BPh)	100 µg/L	PA/Fe^3+^	10.5 mg/L	10^−3^ mol/L	3.0	90 min	95	[88]
3-(4-methylbenzylidene) camphor (4MBC)	100 µg/L	PA/Fe^3+^	10.5 mg/L	10^−3^ mol/L	3.0	90 min	95	[88]
Oxybenzone (OXB)	100 µg/L	PA/Fe^3+^	10.5 mg/L	10^−3^ mol/L	3.0	90 min	95	[88]
N,N-Diethyl-m-toluamid (DEET)	43.73–182.76 µg/L	UV/PAA	0.1425 mL PAA 16%	not reported	not reported	5–10 min	70–100	[98]

**Table 8:** Summary of studies on the use of various activators for PAA activation in pesticides removal.

Pesticides	Pesticides Conc.	Processes	PAA Conc.	Catalyst Dosage/ Activator	pH	Reaction Time	Removal Efficiency (%)	Ref.
Atrazine (AZT)	50 µg/L	UV/PAA	3.2 mg/L	UV 2.3 mW/cm^2^	7.2	not reported	not reported	[78]
	5 µM	(CoFe_2_O_4_)/PAA	0.8 mM	0.5 g/L	7.0	10 min	9.2	[83]
Mecoprop (MCPP)	50 µg/L	UV/PAA	3.2 mg/L	UV 2.3 mW/cm^2^	7.2	not reported	not reported	[78]
Diazinon (DZN)	50 µg/L	UV/PAA	3.2 mg/L	UV 2.3 mW/cm^2^	7.2	not reported	not reported	[78]
Endosulfan I (ESI)	100 µg/L	PA/Fe^3+^	10.5 mg/L	1 mM	3.0	90 min	95	[88]
Endosulfan II (ESII)	100 µg/L	PA/Fe^3+^	10.5 mg/L	1 mM	3.0	90 min	95	[88]

**Table 9:** Summary of studies on the use of various activators for PAA activation in industrial chemicals removal.

Industrial Chemicals	ICBs Conc.	Processes	PAA Conc.	Catalyst Dosage/ Activator	pH	Reaction Time	Removal Efficiency (%)	Ref.
Orange G	0.05 mM	Co_3_O_4_/PAA	0.5 mM	0.1 g/L	7.0	90 min	100	[100]
Bisphenol A (BPA)	15 µM	Co^2+^/PAA	100 µM	10 µM	7.1	30 min	87.7	[56]
	43.8 µM	I^−^/PAA	500 µM	100 µM	3	10 min	100	[101]
	0.02 mM	CNT/PAA	0.25 mM	0.2 g/L	7.0	20 min	96.4	[102]
	10 µM	Co^2+^/PAA	100 µM	10 µM	3.5	30 min	Effective removal	[85]
	30 µM	UV/PAA	0.2 mM	UV 3.8 mW·cm^−2^	3.5	60 min	94	[103]
	1 µM	Fe^3+^/SAc/ PAA	5 mM	0.5 mM Fe^3+^ 1 mM SAc	6.0	10 min	90	[104]
Mix of 10 bisphenols	1 µM	NiCo_2_O_4_/ PAA	7 mM	115 mg/L	7.0	10 min	99.9	[105]
Phenol (Ph)	10 µM	Co^2+^/PAA	0.4 mM	0.05 mM	3–7	10 min	-	[106]
	10 µM	MK-AAFs-Co^2+^/PAA	1.2 mM	20 g, 5.6–10 mm	7.4	15 min	97	[107]
	0.02 mM	CNT/PAA	0.25 mM	0.2 g/L	7.0	20 min	57.6–100	[102]
Rhodamine B (RhB)	5 µM	(CoFe_2_O_4_)/ PAA	0.8 mM	0.5 g/L	7.0	10 min	95	[83]
Bisphenol AF (BPAF)	5 µM	(CoFe_2_O_4_)/ PAA	0.8 mM	0.5 g/L	7.0	10 min	50.6	
2-Chlorophenol (2-CP)	5 µM	(CoFe_2_O_4_)/ PAA	0.8 mM	0.5 g/L	7.0	10 min	79.1	
Aniline	5 µM	(CoFe_2_O_4_)/ PAA	0.8 mM	0.5 g/L	7.0	10 min	70.1	
4-chlorophenol (4-CP)	0.02 mM	CNT/PAA	0.25 mM	0.2 g/L	7.0	20 min	57.6–100	[102]
4-nitrophenol (4-NP)	0.02 mM	CNT/PAA	0.25 mM	0.2 g/L	7.0	20 min	57.6–100	
2-methoxyphenol (2-MOP)	0.02 mM	CNT/PAA	0.25 mM	0.2 g/L	7.0	20 min	57.6–100	
2,4-dichlorophenol (2,4-DCP)	0.02 mM	CNT/PAA	0.25 mM	0.2 g/L	7.0	20 min	57.6–100	
Methyl phenyl sulfoxide (PMSO)	100 µM	Co^2+^/PAA	100 µM	10 µM	3.5	30 min	-	[85]
4-nitrophenol (4-NP)	100 µg/L	PA/Fe^3+^	10.5 mg/L	1 mM	3.0	90 min	95	[88]
4-*n*-nonylphenol (4NP)	100 µg/L	PA/Fe^3+^	10.5 mg/L	1 mM	3.0	90 min	95	[88]
Perfluorooctanoic acid (PFOA)	10–50 µM	Co^2+^/PAA	1–5 mM	10–100 µM	3–5	60–120 min	40–70	[108]
	10–100 µM	Fe^2+^/PAA	1–10 mM	0.1–1 mM	3–6	60–180	30–60	
	10 mg/L	CaCoO_3_/ PAA	1–5 mM	0.1–0.3 g/L perovskite	6–7	60–120	60–80	[109]

**Table 10:** Influence of typical components present in the aqueous matrix on degradation efficiency for different activation methods.

Matrix Component	Main effect on Degradation Efficiency	Mechanistic Role	Dependence on Activation System
HCO_3_^−^/CO_3_^2−^	In case of efficiency occurrence; selectivity shift possible	HO• scavengers → CO_3_^•−^ formation (less reactive but selective)	Strong effect in radical-driven systems (e.g., metal/PAA, UV/PAA)
Cl^−^	Inhibiting or modifying the pathway	Reaction with HO• and CH_3_COOO• → Cl•, Cl_2_^•−^, ClOH^•−^	Strong in Fe^2+^/PAA, spinel catalysts (e.g., NiCo_2_O_4_-PAA)
SO_4_^2−^	Negligible impact	Chemically inert towards ROS under typical conditions	Minimal across systems
NO_3_^−^	In UV systems, slight enhancement	Photolysis → HO• generation	Mainly relevant in UV/PAA
NOM	Strong inhibition	Radical scavenging, metal complexation, UV protection, site blocking	Universal inhibitory effect; strongest in photocatalysis and metalocatalysis

**Table 11:** Characteristics of the main radicals and reactive species formed in PAA-based AOPs.

Radical Species	Symbol Formula	Role and Source of Formation
Acetyloperoxyl radical	CH_3_COOO•	Dominant radical in Co/PAA and CoFe_2_O_4_/PAA systems; major oxidant in cobalt activated PAA reactions
Hydroxyl radical	HO•	Formed during photolysis of PAA and H_2_O_2_ and in Fe^2+^/PAA systems; highly reactive and non-selective oxidant
Acetyloxyl radical	CH_3_C(O)O•	Generated during PAA photolysis and in reactions between Co^2+^ and PAA; transient organic radical undergoing rapid decomposition
Singlet oxygen	^1^O_2_	Produced in UV-C/PAA, UV-C/NaClO and UV-C/PMS processes; contributes to the oxidation of organic compounds
Superoxide radical	O_2_^•−^/HO_2_•	Formed mainly from the decomposition of CH_3_C(O)OO•, less reactive than HO•
Reactive chlorine species	Cl•, Cl_2_^•−^, ClOH^•−^	Generated in UV-C/NaClO systems; involved in chlorination and oxidation processes
Hypobromous acid	HOBr	Formed in PAA/Br^−^ systems; responsible for bromination of organic micropollutants

## Data Availability

No new data were created or analyzed in this study. Data sharing is not applicable to this article.
